# Recent advances in 2D material-based phototherapy

**DOI:** 10.3389/fbioe.2023.1141631

**Published:** 2023-03-03

**Authors:** Yi Tan, Haider Mohammed Khan, Bilal Ahmed Sheikh, Huan Sun, Hui Zhang, Jie Chen, Dingming Huang, Xinmei Chen, Changchun Zhou, Jianxun Sun

**Affiliations:** ^1^ State Key Laboratory of Oral disease, National Clinical Center for Oral Diseases, West China Hospital of Stomatology, Sichuan University, Chengdu, China; ^2^ Department of Cariology and Endodontics, West China Hospital of Stomatology, Sichuan University, Chengdu, China; ^3^ Department of Orthopedics, West China Hospital, Sichuan University, Chengdu, China; ^4^ National Engineering Research Centre for Biomaterials, College of Biomedical Engineering, Sichuan University, Chengdu, China; ^5^ Department of Orthodontics, West China Hospital of Stomatology, Sichuan University, Chengdu, China; ^6^ Department of Pediatric Dentistry, West China School of Stomatology, Sichuan University, Chengdu, China

**Keywords:** 2D materials, photothermal therapy, photodynamic therapy, cancer therapy, antibacterial therapy

## Abstract

Phototherapy, which generally refers to photothermal therapy (PTT) and photodynamic therapy (PDT), has received significant attention over the past few years since it is non-invasive, has effective selectivity, and has few side effects. As a result, it has become a promising alternative to traditional clinical treatments. At present, two-dimensional materials (2D materials) have proven to be at the forefront of the development of advanced nanomaterials due to their ultrathin structures and fascinating optical properties. As a result, much work has been put into developing phototherapy platforms based on 2D materials. This review summarizes the current developments in 2D materials beyond graphene for phototherapy, focusing on the novel approaches of PTT and PDT. New methods are being developed to go above and beyond conventional treatment to fully use the potential of 2D materials. Additionally, the efficacy of cutting-edge phototherapy is assessed, and the existing difficulties and future prospects of 2D materials for phototherapy are covered.

## 1 Introduction

As nanotechnology has rapidly advanced in recent years, researchers have begun exploring nanomaterials’ potential applications in a wide range of biomedical disciplines (Xie et al., 2010; Choi et al., 2012; Shi et al., 2017; Zhang Y et al., 2021). Most notably, 2D materials are rapidly becoming an essential category of nanomaterials for technological advancement (Nicolosi et al., 2013; Hu et al., 2019), which are self-supporting nanosheets with thicknesses of one to several atomic layers (<100 nm) (Novoselov et al., 2004; Chhowalla et al., 2015; Zheng et al., 2021). Since dimensionality is one of the deciding elements for the performance of nanomaterials, the huge surface area, small thickness, flexible composition, and easy modification of 2D materials set them apart from 0D and 1D materials (Novoselov, 2011; Young et al., 2012; Peng et al., 2018; Zhang et al., 2018; Murugan et al., 2019). Due to their appealing characteristics, 2D materials have attracted significant attention from those interested in their potential uses in the fields of device manufacture, environmental cleanup, energy storage, and energy transmission (Li et al., 2011; Bolotsky et al., 2019; Murugan et al., 2019; Hao et al., 2020).

Since the important research that discovered graphene in 2004 (Nicolosi et al., 2013), several investigations into new classes of 2D materials have been undertaken as a direct result of the successful use of graphene and its derivatives, such as nanoelemental nanosheets (Xenes), transition metal carbides, nitrides and carbonitrides (MXenes), transition metal dichalcogenides (TMDs), transition metal oxides (TMOs), layered double hydroxides (LDHs), metal-organic frameworks (MOFs), and Egyptian blue class (XCuSi_4_O_10_), which are generally fabricated through the top–down approach (mechanical exfoliation and liquid phase exfoliation) and bottom–up approach (chemical vapor deposition, pulsed laser deposition, etc.) (Chen et al., 2018, Chen et al., 2022; Mei et al., 2018; Gong et al., 2020; Liang et al., 2020; Li J et al., 2021; Yi et al., 2021; Zheng et al., 2021; Dong et al., 2022; Liu et al., 2023). With specific nanosheet properties, fascinating biocompatibility, and degradability, 2D materials have revealed promising prospects in biomedical applications (Chimene et al., 2015; Wang and Cheng, 2019). For instance, since 2D materials have very high surface-to-volume ratios, they have an outstanding capacity for loading drugs and genes, which makes them suitable for use as delivery platforms based on the nanoscale (Chimene et al., 2015; Chen W et al., 2017; Lu et al., 2021). Additionally, encouraging module values and low toxicity reveal promise for strengthening the mechanical characteristics of biomedical materials at very low concentration, which is essential for tissue regeneration (Chimene et al., 2015; Banerjee, 2018; Zheng et al., 2021). Due to their unique optical properties, they have been applied in various molecular imaging techniques (Eom et al., 2020).

Recently, the optical properties of biomedical materials have been further exploited, with 2D materials being considered alternative agents for phototherapy. This includes photothermal therapy (PTT) and photodynamic therapy (PDT), in which the energy of photons excited by light illumination is used to generate heat and reactive oxygen species (ROS) to carry out the therapy. A quick rundown of why this picture agent is better than the norm is as follows: 1) strong and wide absorption from the ultraviolet (UV) to the near-infrared (NIR), which may be controlled by changing the material’s thickness. 2) Quantum yields and photostability are both relatively high. 3) Photosensitizers and medicines can be added to facilitate synergistic treatment (Tao et al., 2019; Wang and Cheng, 2019; Liu Q et al., 2020).

**SCHEME 1:**
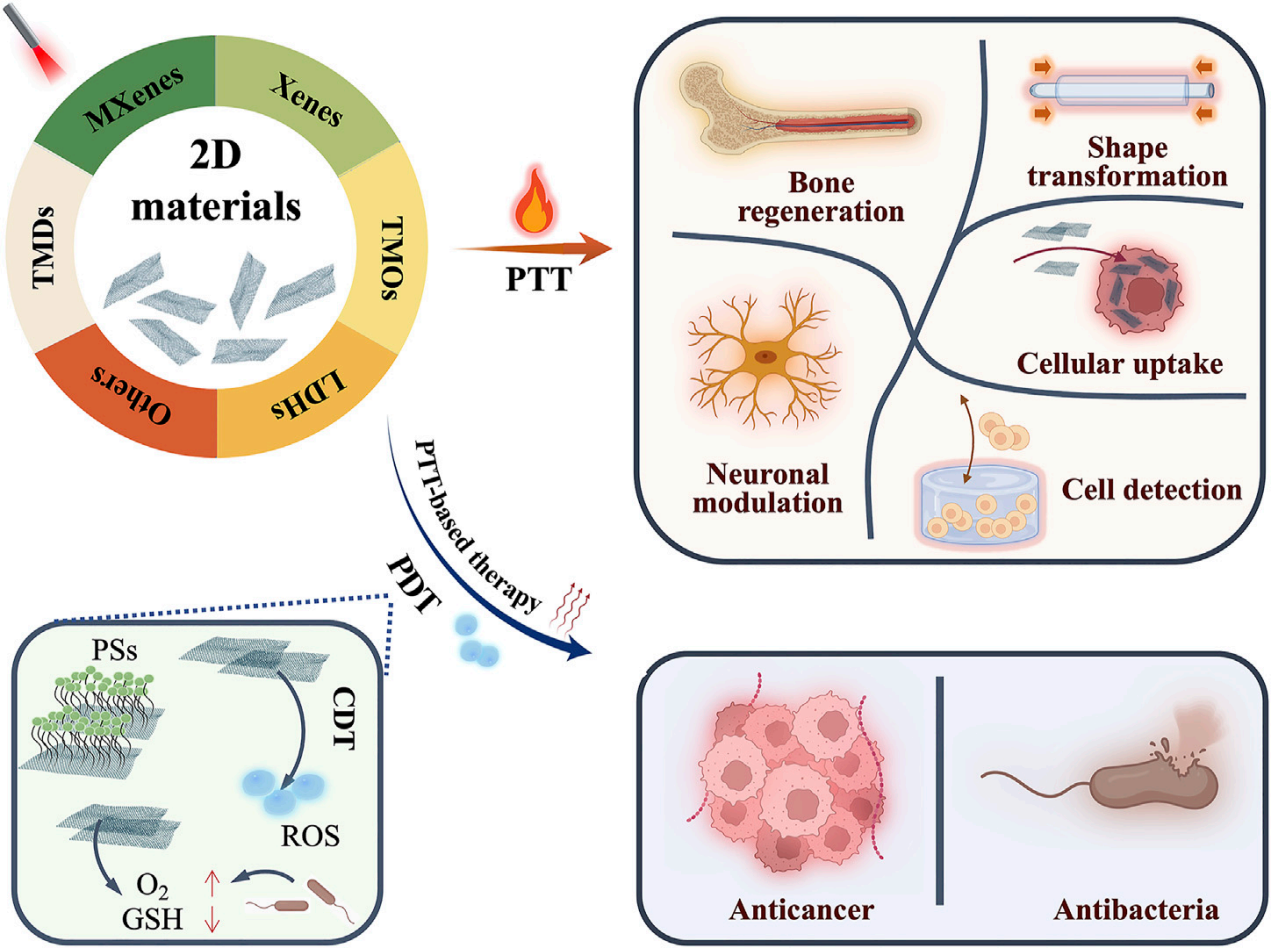
Schematic illustration for biomedical applications of phototherapy based on 2D materials.

The use of 2D materials in cancer phototherapy has been extensively documented in various published works until now. However, few evaluations have looked at the use of 2D materials in PTT for conditions other than cancer and diverse strategies of PTT and PDT. This review aims to shed light on the newest developments in 2D materials beyond graphene for PTT and PDT and develop novel therapeutic techniques. Several biomedical applications in cancer, bacterial infection, bone regeneration, and others are presented, and some advances in phototherapy are shown to demonstrate the various ways in which phototherapy is being used more widely (Scheme 1). The review was written to generate enthusiasm for phototherapy mediated by 2D materials.

## 2 PTT

The use of photothermal agents (PTAs) to convert photon energy to thermal energy under light irradiation and thus create local hyperthermia for clinical purposes is a relatively novel non-invasive treatment method showing promising results in recent years (Liu X et al., 2019; Hu et al., 2020; Ma K et al., 2021; Sutrisno et al., 2021; Xu Q et al., 2021). Due to their unusual optical property and distinctive nanosheet structure, 2D materials are seen as potentially important options for photothermal treatments. This is because they may achieve temporal and spatial control of the generated heat (Wang X et al., 2019; Liu Q et al., 2020; Wang S et al., 2020; Chang et al., 2022).

### 2.1 Photothermal-mediated cancer and bacterial infection therapy

Recent advances in PTT among 2D materials for anticancer and antibacterial treatments are introduced with a brief illustration of several typical examples. This is because extensive research on PTT for cancer and infectious diseases has been conducted in most 2D materials, showing similar treatment strategies.

#### 2.1.1 Single-mode PTT in the first NIR window (NIR-I)

PTT is now widely used to treat cancerous tumors. Hyperthermia kills tumor cells due to its destructive effects on DNA, cell membranes, and mitochondria, inhibiting metabolism and denaturing proteins (Yuen et al., 2000; Huang et al., 2019; Liu Y et al., 2020). The nanoscale allows 2D materials to concentrate at the tumor site through an increased permeability and retention (EPR) effect. In addition, the use of 2D materials as photothermal agents in PTT displays extraordinary results for tumor ablation because of their high photothermal conversion efficiency and their exceptional light-absorbing capacity (Lin et al., 2018; Dai et al., 2020; Kumar et al., 2021).

**FIGURE 1:**
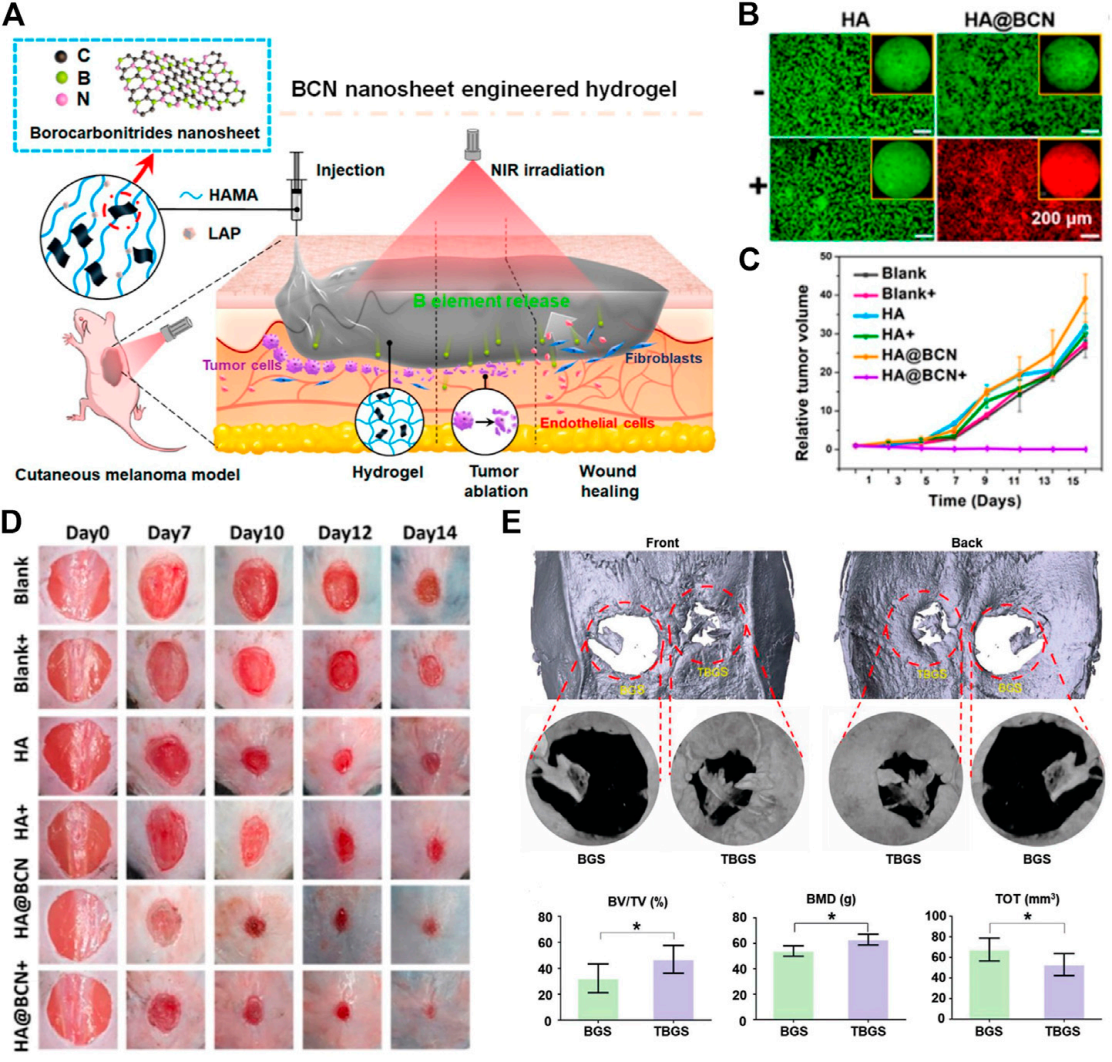
**(A)** Schematic illustration of the therapeutic strategy for melanoma based on HA@BCN. **(B)** Live/dead staining of B16F10 cells in different groups. **(C)** Growth of tumor volume in different treatment groups. **(D)** Digital photographs of skin regeneration during the repair period in different groups. (Adapted with permission from Zhao et al., 2022. Copyright 2022 American Chemical Society). **(E)** Micro-CT images and value of BV/TV, BMS, and TOT at 24 weeks after implantation in different groups. (Adapted with permission from Pan et al., 2020. Copyright 2019 Wiley-VCH).

The rising incidence of melanoma has refocused research on PTT, which places a premium on the bioactivity of PTAs. Releasing the boron element, a unique borocarbonitride (BCN), has shown a considerable advantage in treating cutaneous injuries (Zhao et al., 2020). The melanoma-curing HA@BCN was created by combining hyaluronic hydrogel (HA) with 2D borocarbonitride (Figure 1A). The intriguing photothermal characteristics of HA@BCN nanosheets, whose temperature control is dependent on BCN concentration and laser power density, are made possible by the high conversion efficiency of BCN nanosheets. Hyperthermia caused by laser irradiation increased cancer cell mortality to over 80% (Figure 1B), which eventually resulted in tumor inhibition (Figure 1C). The advantages of HA and BCN in tissue regeneration following PTT accelerated the repair of residual skin defects (Figure 1D) (Zhao et al., 2022). Since PTT processes for cancer are largely the same across different 2D materials, PTT mediated by 2D materials seems to be effective across the board for cancer treatment. However, due to their unique chemical compositions, individual nanosheets could show distinctive responses to different tissues, such as BCN (Zhao et al., 2022) for skin regeneration and black phosphorus (BP) (Yang X et al., 2018) and Ti_3_C_2_ (Pan et al., 2020) (Figure 1E) for bone regeneration. To determine which 2D materials are best for treating certain malignancies, further study is needed to determine if PTT mediated by distinct nanosheets has distinguishing effects on tumors.

When applied to bacterial infections, thermal stimuli kill bacteria by rupturing their membrane, which allows nucleic acid and enzymes to flow out and degrade. Hyperthermia induction also destroys the biofilm structure, removing the barrier and leaving the bacterial matrix susceptible to both thermal stimuli and antibiotics (Huo et al., 2021; Xu X et al., 2021). Since 2D materials have a flake shape, the sharp edges of the nanosheets cause extensive physical damage to the bacterial structure, which may be combined with PTT to improve antibacterial therapies (Nelson et al., 1986; Perreault et al., 2015; Wu et al., 2015).

**FIGURE 2:**
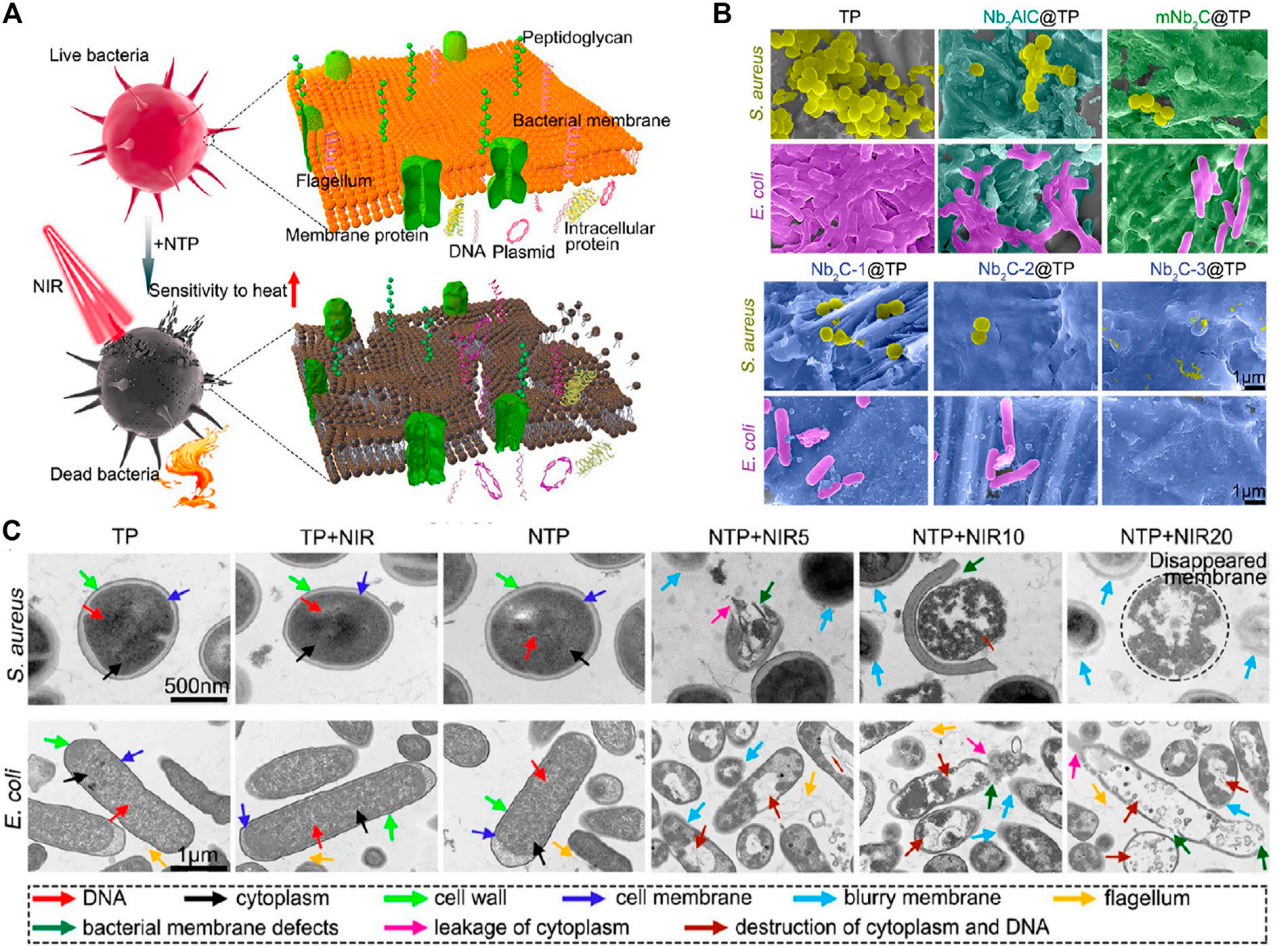
**(A)** Schematic illustration of the therapeutic strategy for bacterial infection based on Nb_2_C@TP. **(B)** SEM images of biofilm-resistance performance in different groups. **(C)** TEM images of antibacterial performance in different groups. (Adapted with permission from Yang et al., 2021. Copyright 2021 American Chemical Society).

The concentration-dependent antibacterial activity of Ti_3_C_2_ was shown in MXene, and its antibacterial effect was significantly enhanced when exposed to laser light, even at very low Ti_3_C_2_ concentrations (Wang et al., 2021a). Further explanation of the dual-mode method is provided in Figure 2A. Sharp Nb_2_C, coated on a titanium plate, showed nanosheet size-dependent disruption of biofilm development (Figure 2B). Activation of the Agr quorum sensing system also caused disruptions in the expression of genes involved in biofilm formation, resulting in more efficient biofilm suppression and disintegration. Furthermore, the susceptibility of bacteria to heat stimuli was elevated by incomplete biofilms, which explains why phototherapy had such a devastating effect on bacterial walls and membranes (Figure 2C) (Yang et al., 2021). From the perspective of synergistic effects, it is clear that using 2D materials in conjunction with PTT lowers the necessary temperature for phototherapy and the concentration needed to physically damage nanosheets, both of which lead to less damage to the surrounding tissue and less toxicity to the organism in antibacterial treatments.

#### 2.1.2 Single-mode PTT in the second NIR window (NIR-II)

Although NIR-II (1000–1350 nm) has a larger penetration depth of tissue, much of the existing research studies on nano-photosensitive agents still focus on NIR-I (750–1000 nm). In addition, advancements in this sector need to be improved by the scarcity of materials with high light absorption and photothermal conversion efficiency in the NIR-II range. Because of this, efforts are being made to find ways to overcome this barrier by using 2D materials with exceptional optical performance (Sun et al., 2018; Huang J et al., 2020).

In this context, Nb_2_C-PVP showed that the 1063-nm laser penetrated deep tissue with less energy loss and scattering than the 808-nm laser (Lin et al., 2017). For PTT in the NIR-I and NIR-II regions, another research synthesized PtAg nanosheets through a wet-chemistry approach. PtAg showed strong temperature-increasing ability and excellent photostability under 785 nm and 1064 nm irradiation, qualifying it as a therapeutic agent for tumor ablation in dual bio-windows (Zhang X et al., 2021). Analyses of these bio-windows have highlighted the different benefits of NIR-II. As NIR-II light is weak in producing tissue self-heating, it is more acceptable for use in the human body, and further inquiry in PTT is predicted to focus on NIR-II. To effectively remove tumors in the NIR-II bio-window, a modified CaCO_3_–PCL scaffold was coated with CaCuSi_4_O_10_ nanosheets and used in treating osteosarcoma. Thermal stimuli may be the major source of PTT-induced cell apoptosis because they disrupt genetic activity associated with the mitotic cell cycle, the response to nucleic acid damage, cell migration, and other processes (He et al., 2021).

#### 2.1.3 PTT combined with drug therapy

Previous research evaluated the anti-cancer efficacy of PTT alone with that of PTT coupled with chemotherapy, showing that the latter was more effective. This is because PTT alone only serves a brief role during irradiation (Liu et al., 2017; Han X et al., 2018). Similarly, the antibacterial impact of a single mode of PTT was not sufficient to completely eradicate the illness since residual germs would keep multiplying (Yang Y et al., 2022). 2D materials have been studied for their potential as a delivery platform in phototherapy due to their unique nanosheet structure, which has a high surface area and is equipped with adequate anchoring sites for therapeutic compounds. Synergistic treatment caused by photothermal stimulation may be applied more easily due to the enhanced performance of nanocarriers (Lin et al., 2018).

Currently, there is a system in place for the controlled delivery of medications. Doxorubicin (DOX) was added to 2D Ti_2_N after it was treated with soybean phospholipid (SP). Once the organosilica shell was prepared, cisplatin and the therapeutic substance were loaded. On arrival at the tumor site, H^+^ reduced the connection and facilitated the release of cisplatin, whereas organosilica shell degradation set in. DOX revealed pH-responsive and temperature-responsive release that could be readily regulated by light stimuli as the restriction shell eventually disintegrated (Li D et al., 2021). PTT’s curative effects were further solidified by the realization of a synergistic platform in bacterial infection, wherein nanosheet-induced thermal stimuli revealed a strong lethal effect on bacteria while simultaneously promoting the release of antibiotics that distinctively strengthened the antibacterial activity (Guo et al., 2020; Xu T et al., 2021).

**FIGURE 3:**
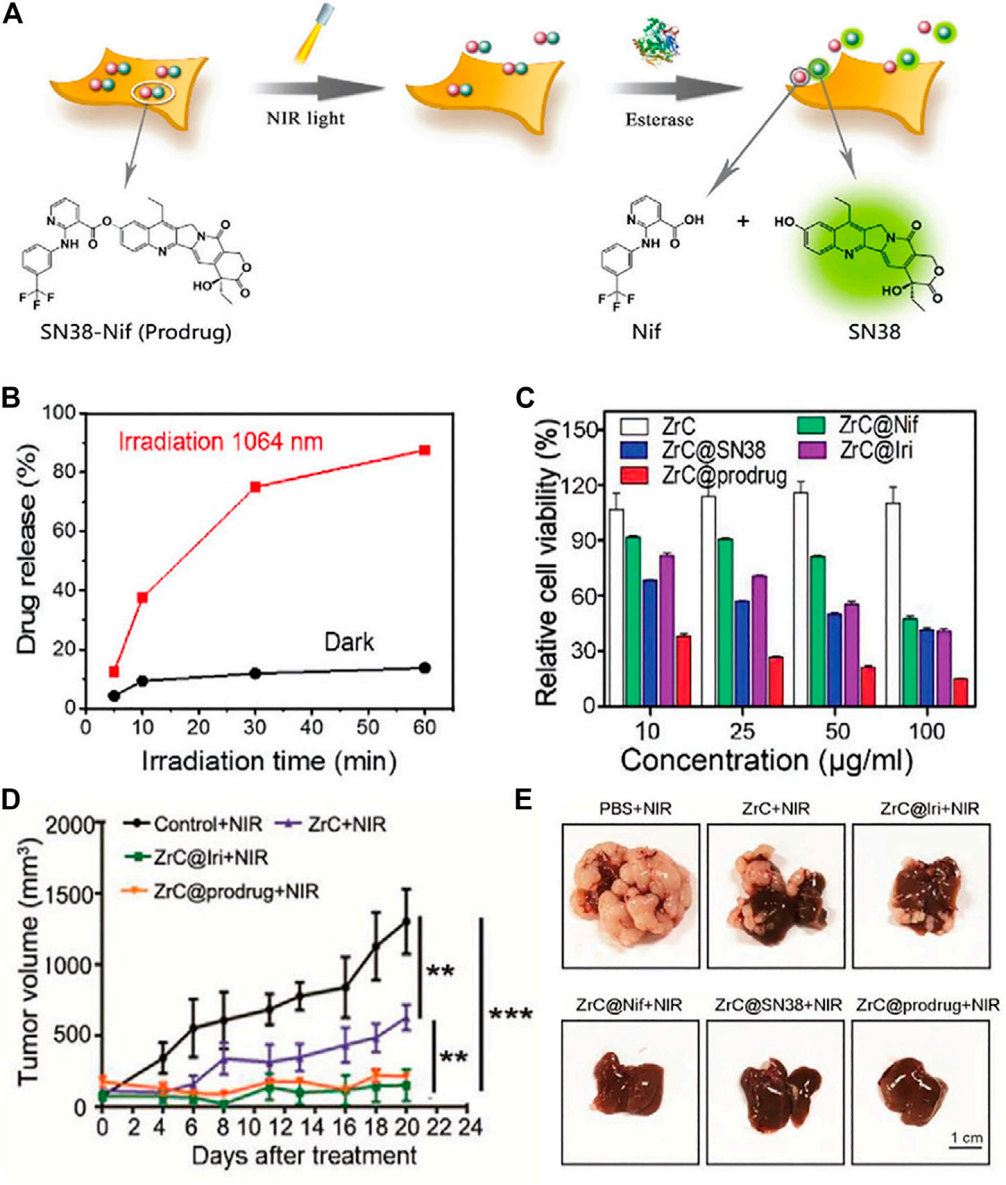
**(A)** Schematic illustration of release and activation of SN38-Nif based on ZrC@prodrug. **(B)** Drug release percentage with and without irradiation. **(C)** Relative cell viability of SMMC-7721 in different groups. **(D)** Growth of tumor volume in different treatment groups. **(E)** Digital photographs of tumor after treatments in different groups. (Adapted with permission from Liu S et al., 2020. Copyright 2020 Wiley-VCH).

Interestingly, although photothermy does increase drug release following heat stimulation, it also unexpectedly hastens the diffusion of medication that is more prone to disseminate into normal tissues. Prodrugs are novel drugs developed to improve a drug’s stability in the body and lessen its side effects. Prodrugs, which only become effective as drugs in a tumor’s unique environment, are a potentially useful alternative to conventional therapies (Chen D et al., 2021; Srinivasulu et al., 2021). After laser irradiation, ZrC nanosheets resulted in the production of the prodrug SN38–Nif (Figures 3A, B). To further increase tumor inhibition in PTT, the released prodrug was converted into the chemotherapeutic medicines SN38 and Nif in the presence of overexpressed carboxylesterase in tumor cells (Figures 3C–E). Also, since normal cells do not produce esterase, the prodrug remains unchanged, protecting healthy tissue from unwanted drug effects (Liu S et al., 2020). Several disorders have responded well to PTT’s treatment approach. Synergistic therapies focus on the precise and toxic control of drug release in terms of release speed, release place, and release sequence, in contrast to the improved ability to destroy the tumor and bacterial cells. In addition, anticancer medications and antibiotics for wound fixing may be administered along with therapeutic agents for tissue healing as part of a synergistic approach.

#### 2.1.4 PTT combined with other therapeutic agents

Various studies have shown that the unique 2D structure of nanosheets makes them ideal for drug loading, and the functional groups connected to the vast surface area may be used to anchor other bioactive molecules. PTT-mediated release of ferrous ions that might create ROS for fighting tumors by PTT–chemodynamic synergistic treatment was established in an original study in which Ti_3_C_2_ stored ferrous ions into nanosheets through electrostatic adsorption of hydrophilic groups (Wu et al., 2021a).

Though the use of antibiotics in synergistic treatment boosts PTT’s therapeutic efficacy, it would run against PTT’s goal of avoiding the development of antibiotic-resistant microorganisms. Remarkably, antimicrobial peptides are included to combat bacterial infections, which exhibit little resistance tendency (Gupta et al., 2019). Unlike conventional antibiotics, the natural antimicrobial peptide ϵ-poly-L-lysine (ϵ-PL) was maintained on BP nanosheets and was active during PTT. Since the surface of ϵ-PL is positively charged, BP@ϵ-PL would bind to the negatively charged bacterial membrane, contributing to the destruction of the membrane’s structure due to photothermy (Fu et al., 2022). Infections carried on by resistant strains may be treatable because of the synergistic effects of PTT’s rapid and potent antibacterial activity and the antimicrobial peptides’ persistent ability to adhere to and damage bacteria. Additionally, PTT-antimicrobial peptide treatment may accomplish the goal of synergistic therapy requiring sustained antibacterial ability without the adverse effects of antibiotics.

A photothermal-triggered method was also observed in another facet of gas treatment. To improve anticancer and antibacterial therapy, nitric oxide (NO) donors such as S-nitrosothiol and BNN6 loaded on 2D nanosheets have allowed precise regulation of (NO) releasing in response to light stimuli (Yang C et al., 2020; Liu S et al., 2021). Nanosheets are expected to mediate the concentration and release of gas at target areas, resolving the issues of unregulated diffusion and low solubility that hamper gas therapy.

#### 2.1.5 PTT combined with immunotherapy

Currently, immunotherapy has made significant progress in the battle against aggressive and distant tumors, combining chemotherapy mixed with PTT for metastasis and recurrence of cancer (Xu et al., 2013). Immunotherapies, including chimeric antigen receptor T cell (CAR-T) therapies, immunological checkpoint blockade (ICB) therapies, and tumor vaccines, have shown promising results in identifying and killing cancer cells with durable anticancer responses by stimulating the body’s immune system (Han C et al., 2018; Helmink et al., 2018; Lecoq et al., 2022). Despite its potential, immunotherapy is limited in its use because of patient-to-patient variance, poor antitumor efficacy, and coexisting adverse effects (Wei et al., 2019; Fisher et al., 2020; Hu et al., 2022). PTT has been used in conjunction with immunotherapy to improve cancer therapies in recent years. Evidence from the past suggests that PTT will have a secondary effect on tumors by activating the immune system *via* producing damage-associated molecular patterns (DAMPs) and tumor-associated antigens (TAAs) (Ma et al., 2019; Xu and Liang, 2020). In contrast, 2D materials laden with immunological adjuvants and immune checkpoint inhibitors serve as excellent nanocarriers, which may make up for the modest immune activity caused by PTT (Ming et al., 2020; Wan et al., 2020; Fang et al., 2021).

Immunological adjuvants R837 were added onto BP nanosheets to boost immune performance since the stimulation of dendritic cells (DCs) generated by PTT was insufficient for cancer immunotherapy. Signal cytokine (TNF-α, IL-6, and IL-12) production and DC maturation were improved by adding R837 to the immunostimulation protocol compared with the use of PTT alone (Wan et al., 2020). In comparison, 2D material-mediated PTT also enhanced the ability of immunological adjuvants to accomplish their tasks. Cytosine–phosphate–guanine (CpG) showed an improved ability to enter cells through endocytosis of nanosheets when placed on 2D Pd, which can enhance CpG’s capacity to mediate an immune-related pathway. PTT combined with CpG was more effective in increasing surface molecule expression (CD86 and CD80) and immune cell proliferation than either PTT or CpG individually (Ming et al., 2020).

**FIGURE 4:**
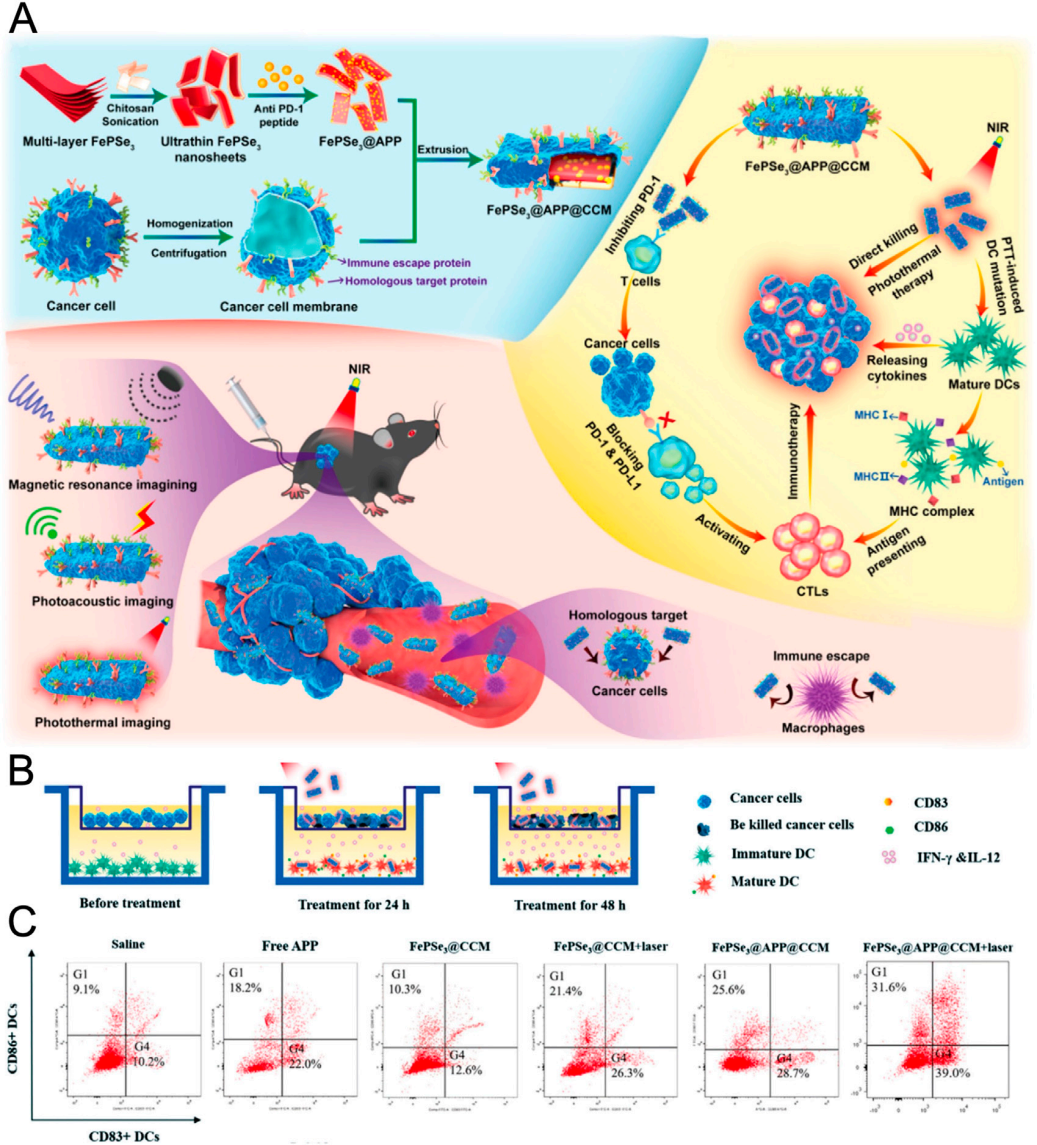
**(A)** Schematic illustration of fabrication of FePSe_3_@APP@CCM and the strategy of combined PTT immunotherapy. **(B)** Schematic illustration of DC maturation during the treatment. **(C)** Flow cytometry analysis of DC maturation in different groups. (Adapted with permission from Fang et al., 2021. Copyright 2020 Wiley-VCH).

Anti-programmed death-1 peptide (APP) was fused with FePSe_3_ nanosheets for use in immune checkpoint treatment (Figure 4A). When bound to FePSe_3_, APP blocks PD-1’s suppressive effect on the immunological response, hence enhancing the PTT-induced T-cell-related immunoreaction (Figures 4B, C) (Fang et al., 2021). Interestingly, 2D nanosheets might trigger beneficial humoral immunity in an infection context, demonstrating potent suppression of the bacterial biofilm by controlling the production of costimulatory molecules and antigen presentation (Yang C et al., 2022). Furthermore, it was thought that heat stimulation is effective in attracting immune cells to infection sites for reducing inflammation (Zhao et al., 2019). The key advantages of nanosheets suggest that PTT mediated by 2D materials may exhibit extraordinary immunological activity in antibacterial treatment. Nonetheless, there has yet to be a known study in this area. It is hoped that 2D materials will pave the way for new avenues of investigation into refractory infection by being employed in antibacterial therapies in combination with immunotherapy.

### 2.2 Photothermal-mediated bone regeneration

Several research works have identified additional rare uses of phototherapy in addition to the aforementioned common photo-triggered therapies that most 2D nanomaterials share. Numerous studies over the last several years have shown that 2D materials have a good impact on bone defects owing to their unique physical and chemical features that are helpful for bone regeneration (Yang X et al., 2018; Yang Q et al., 2020). The positive impact of 2D materials on osteogenesis has also been demonstrated by research in immunoregulation linked to metabolic reprogramming (Xue et al., 2018; Du et al., 2022). Although the precise method by which PTT promotes bone regeneration has yet to be elucidated, a few studies have shown that moderate temperature will enable the interchange of nourishment, enhance blood circulation, and stimulate osteogenic differentiation of stem cells. It was also hypothesized that photon-involved gene expression might be manipulated to enhance bone repair during PTT (Wu X et al., 2022). Therefore, there is a huge unrealized potential in 2D material-mediated phototherapy for bone regeneration, which has not been explored due to lack of research exploiting the optical capabilities of nanosheets in bone defects.

Recently, poly (lactic-co-glycolic acid) (PLGA), a substrate appropriate for tissue engineering, was combined with black phosphorus nanosheets (BPs) to create a BPs@PLGA membrane. The proliferation rate of mesenchymal stem cells was shown to increase gradually with increasingly longer duration (0 s, 30 s, and 60 s) of light stimulation after periodic moderate-temperature shocks at 40°C. Overexpression of heat shock proteins (HSPs), which determine a cell’s thermotolerance, has been linked to the fact that cells are unaffected by moderate temperatures. Additionally, upregulated HSPs aided in stem cell differentiation and osteoblast development, which may also be attributed to the increased production of osteogenesis-related proteins (Tong et al., 2019). When hydroxyapatite scaffolds were modified with BPs, a similar result was observed, with both BP nanosheets and mild heat stimulation contributing to rebuilding of bone tissue (Wu et al., 2021b).

Researchers have shown some preliminary success in using PTT for bone repair thanks to a theory based on protein expression. Gene expression is a potential avenue for future research because of its role as an upstream process in defining a distinct route. Poly (ε-caprolactone)/molybdenum disulfide (PCL)/MoS_2_ membranes were fabricated and studied to direct bone regeneration (GBR). Under periodic laser irradiation that caused modest temperature stimuli, the expression of osteogenesis-related genes such as RUNX2, OCN, OPN, ALP, BMP2, and COL1a1 was dramatically increased. HSP gene expression was also upregulated in response to heat stress. By maintaining a temperature of 40°C, cell apoptosis could be prevented, and the beneficial effects of PTT on bone regeneration could be maximized (Ma T et al., 2021). However, more research and analysis are still needed to optimize the sustained time of laser irradiation and the final temperature, striking a balance between bone regeneration and unnecessary tissue damage, even though periodic irradiation for 60 s at a moderate temperature has been proven efficient for osteogenesis, without side effects. In addition, the intrinsic mechanism is not clear enough; additional research is needed to determine the precise role of PTT. This study, however, broke the monopoly of high-temperature treatments in PTT and shed light on the potential of PTT in the treatment of bone defects, while also opening the door to the investigation of mild-temperature phototherapy.

### 2.3 Photothermal-mediated neuronal modulation

Numerous techniques of neural modulation have been established in earlier research, with most of their development driven by the search for modulation with high spatial resolution, which would allow for more in-depth knowledge of complicated neuronal circuitry. To avoid invasive surgery and genetic alteration in electrode implants and optogenetic therapies, recent research has focused on PTT, in which thermal stimuli activate neurons through transient receptor potential channels related to temperature responsiveness. Moreover, nanoscale 2D materials have been used in biomedicine with high resolution precise to subcellular accuracy (Jung and Nam, 2022; Wu Y et al., 2022). Therefore, PTT mediated by 2D materials is of significant academic value in fields of neurology.

**FIGURE 5:**
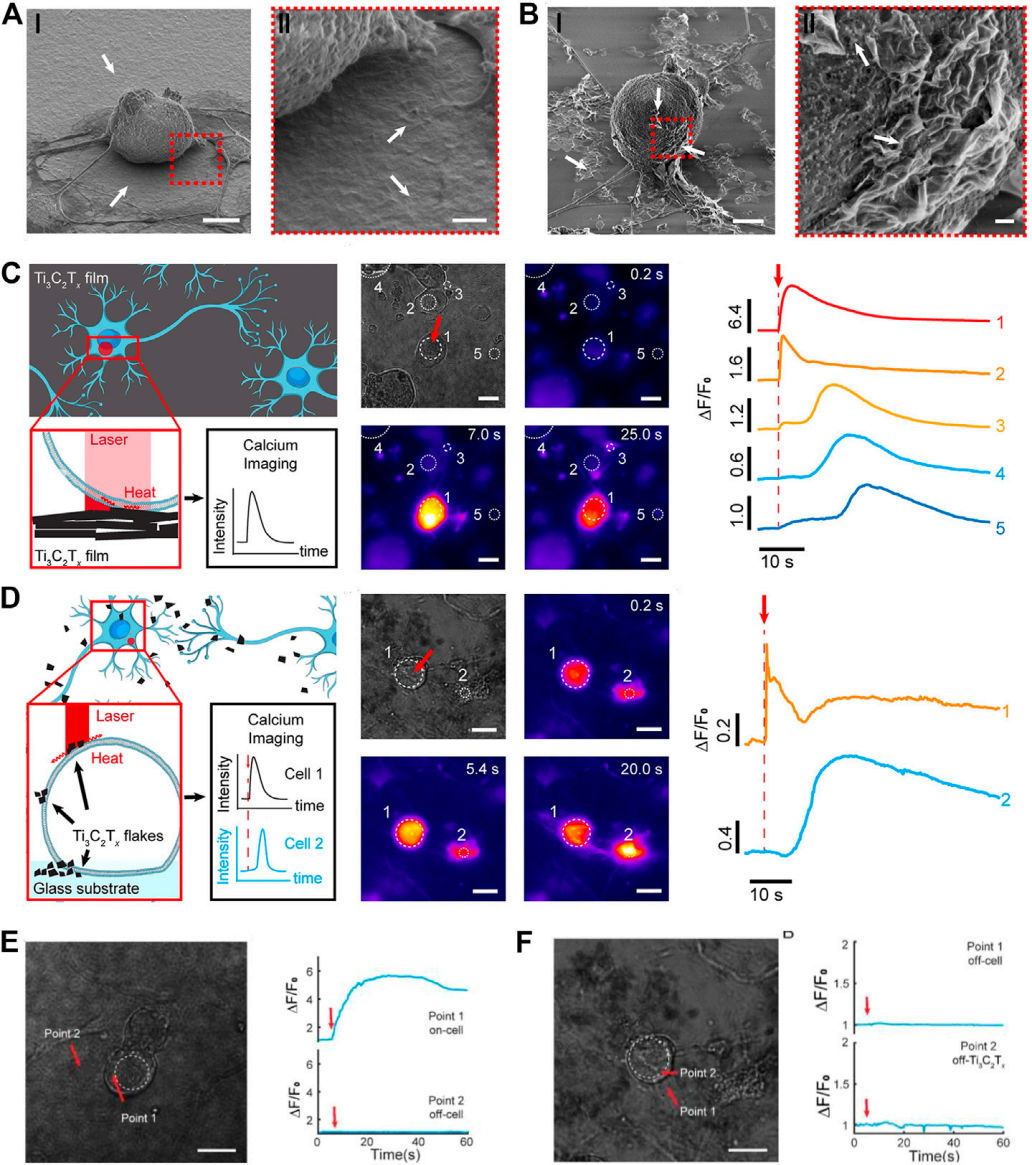
**(A)** SEM images of the interface between Ti_3_C_2_T_X_ films and DRG neurons. **(B)** SEM images of the interface between Ti_3_C_2_T_X_ flakes and DRG neurons. **(C)** Illustration of Ti_3_C_2_T_X_ film-mediated DRG electrical activity modulation. **(D)** Illustration of Ti_3_C_2_T_X_ flake-mediated DRG electrical activity modulation. **(E)** Illustration of selective modulation of DRG neurons mediated by Ti_3_C_2_T_X_ films. **(F)** Illustration of selective modulation of DRG neurons mediated by Ti_3_C_2_T_X_ flakes. (Adapted with permission from Wang et al., 2021a. Copyright 2021 American Chemical Society).

To this objective, Ti_3_C_2_T_x_’s ability to regulate neural activity was studied in an effort to fully exploit the spatial resolution afforded by 2D materials. Neurons from the dorsal root ganglion (DRG) adhere tightly to the Ti_3_C_2_T_x_ films underneath them due to their nanosheet structure (Figure 5A), and flakes distributed throughout the films adhered to the membranes of DRG neurons all over the place (Figure 5B), without affecting cell viability. When the adhesion between Ti_3_C_2_T_x_ and DRG is good enough, it is possible to transmit thermal stimuli from Ti_3_C_2_T_x_ to DRG. Extremely low-energy irradiation with a 625-nm laser would provide thermal stimulation encouraging Ca2+ transients *via* electrical activity (Figures 5C, D). The Ti_3_C_2_T_x_ film directly contacting DRG neurons was crucial for effective neuronal stimulation (Figure 5E). In addition, the subcellular scale of Ti_3_C_2_T_x_ flakes allowed for neuronal modulation with high spatial resolution, as Ca2+ transients could not be triggered unless the laser target was situated in the interference between neurons and nanosheets. This held true regardless of whether the laser aimed at a neuron or a Ti_3_C_2_T_x_ flake (Figure 5F) (Wang et al., 2021a). Understanding the function of neurons, which has long baffled scientists, requires subcellular-level control of where stimuli are sent to neurons. As nanomaterials advance, limitations on neurological studies would be lifted, which would benefit medical imaging and other sectors of medicine that rely heavily on precise spatial positioning.

### 2.4 Photothermal-mediated shape transformation

Another novel aspect of PTT is associated with shape memory polymers (SMPs), which have a special feature of shape recovery under specific external stimulations, including thermal stimuli. The applications of thermal-responsive SMP in medicine are flourishing as it has shown significant potential in biosensors and biological stents (Hao et al., 2021). However, in comparison to light stimuli, local temperature control of SMP needs more precision (Jiang et al., 2006). A significant step toward this goal is provided by 2D nanosheets with exceptional photothermal characteristics, which can convert thermal power into light stimulus control.

**FIGURE 6:**
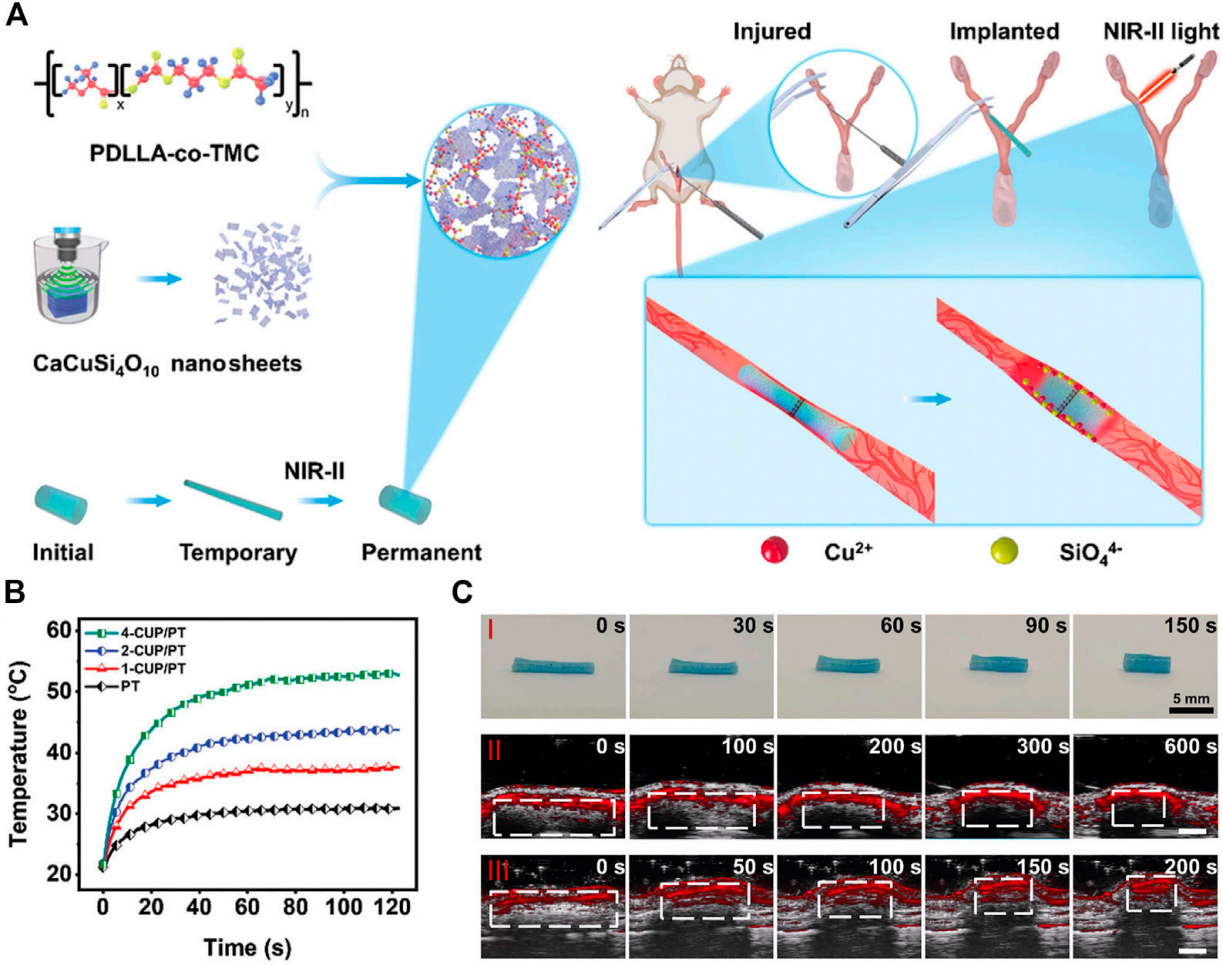
**(A)** Schematic illustration of the therapeutic strategy for IUA prevention based on CUP/PT. **(B)** Photothermal property of PT and CUP/PT under 1064-nm laser irradiation. **(C)** Shape recovery of CUP/PT at different time after irradiation on a desk (I), underneath the skin (II), and inside the isolated uterine lumen of rat (III), respectively. (Adapted with permission from Dong et al., 2022. Copyright 2022 Wiley-VCH).

The cuprorivaite nanosheet and conventional SMP PDLLA-co-TMC (PT) were combined to create a photothermal-sensitive composite CUP/PT for treating intrauterine adhesions (IUAs) (Figure 6A). The critical temperature triggering shape alteration in this PT was slightly higher than body temperature, making it very appropriate for moderate photothermal use in the human body. In addition, 2D cuprorivaite, which is essential for the interaction between light stimulation and shape change, provided CUP/PT with excellent photothermal conversion ability and good photothermal stability (Figure 6B). After being stretched to a longer and thinner pattern at 80°C, CUP/PT tubes were cooled to room temperature to temporarily retain their new form, making the implant procedure easier. As earlier studies have shown, even when subjected to laser irradiation, temperature-dependent SMPs would retain their deformed state for some time without thermal stimuli (Li et al., 2019). Nevertheless, when the SMP was treated with a photothermal agent, the light stimuli were converted into thermal stimuli, resulting in rapid shape restoration of the stretched SMP at implant locations after laser irradiation. Pre-stretched smaller SMPs on IUAs enable easier procedures, and their subsequent recovery to a somewhat broad starting shape allows them an effective barrier against intrauterine adhesion (Figure 6C) (Dong et al., 2022).

The photothermal characteristics of BP and the temperature sensitivity of SMP polyurethane (PU) were successfully combined in an early experiment. After laser irradiation, the diameter of the stretched BP/PU column in the mouse vagina significantly increased to its original size, which was broader enough for fallopian obturation, demonstrating the excellent shape transformation of the PU/BP column under light stimuli and its significant potential in fallopian obturation after laser irradiation (Xie et al., 2018). Based on the available literature, the 2D nanosheet-modified SMP increases the likelihood of stent placement by minimally invasive techniques. Furthermore, depending on the shape transformation performed, investing in better shape compatibility between bone scaffolds and bone defects may be beneficial (Zhang et al., 2022).

### 2.5 Photothermal-mediated cellular uptake

The transfer of chemicals into particular cells, known as cellular uptake, is the deciding element in therapeutic approaches like chemotherapy and gene therapy. There is still a hurdle to be overcome in the absence of a delivery platform capable of loading drugs and genes with high efficiency of cellular absorption (Dong et al., 2018; Liu and Du, 2021). Recently, attempts have been made on nanocarriers to solve this challenge, with an emphasis on 2D materials that show significant possibility. The uptake performance of 2D nanosheets is dependent on their size and concentration, and cells collect them through an energy-dependent internalization mechanism controlled by clathrin (Chen et al., 2016; Tyagi et al., 2017; Wang et al., 2017). However, PTT has recently been utilized in nanomaterials to further improve cellular absorption, demonstrating that heat stimulation may substantially increase cellular membrane fluidity and permeability (Liu Y et al., 2019; Ma et al., 2020). On the other hand, the importance of PTT’s effects on cellular absorption in tumor treatment is often overlooked.

**FIGURE 7:**
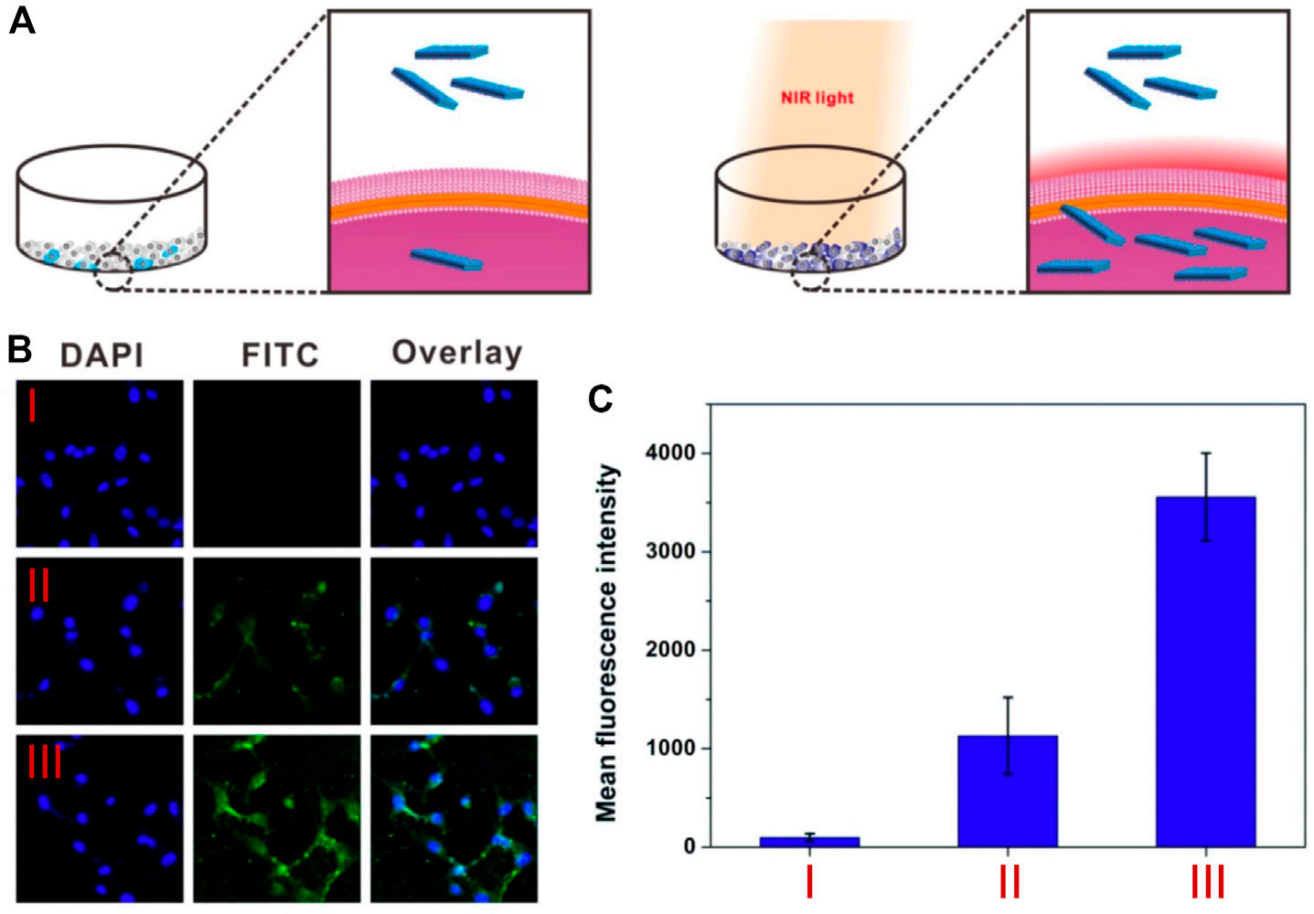
**(A)** Schematic illustration of photothermal-mediated cellular uptake of BP-HAS-PTX. **(B)** CLSM images of U87MG cells incubated with PBS (I), BP-HAS-PTX without laser irradiation (II), and BP-HAS-PTX with laser irradiation (III), respectively. **(C)** Mean FITC fluorescence intensity of U87MG cells in different groups. (Adapted with permission from Wang et al., 2017. Copyright 2017 Ivyspring International Publisher).

Drug delivery systems include BP filled with paclitaxel (PTX). As a result of 2D materials’ inherent absorption capability, cancer cells were able to seize a negligible fraction of the nanosheets (U87MG cells). In response to laser irradiation, BP was heated to 42.5°C ± 0.5°C, which improved cell permeability and allowed a large number of nanosheets to penetrate the cells, while preventing unnecessary damage to the surrounding average cell population (Figure 7A). By combining PTX with nanosheets for delivery into cancer cells, the anticancer impact of chemotherapy might be improved (Figures 7B, C) (Wang et al., 2017). In leukemic cells, laser irradiation also triggered the increase of cell membrane permeability, which increased the cellular absorption of MoS_2_-PEG-CpG nanosheets (Han et al., 2017). Nanosheets laden with drugs can penetrate cells efficiently due to the robust cellular absorption mediated by PTT, which also opens the door to the prospect of highly targeted chemotherapy and local photothermal treatment. Uptake into the site of the illness may also be possible if the vehicle is laden with fluorescent compounds used for diagnostic imaging.

### 2.6 Photothermal-mediated cell detection

As a crucial signal of tumor metastasis and recurrence, circulating tumor cells (CTCs) that spread from the site of the original tumor to the blood circulatory system are of significant importance in cancer diagnosis and evaluation of tumor progression. Compared to conventional biopsies, CTC measurement through liquid biopsy is much less invasive (Dou et al., 2019; Zhou et al., 2022). In the past, nanomaterials have been produced to build nanoplatforms for detecting CTCs, taking advantage of their large surface area and biomolecular size. To a significant extent, nanoparticles’ magnetic properties are used in scientific investigation. As a counterpoint, PTT has received comparatively less attention because of the difficulty of using its photothermal feature in the collection and release of CTCs (Yoon et al., 2013, Yoon et al., 2016; Xu et al., 2020). Due to nanosheet development being in its infancy, PTT is now the subject of research for potential future application.

Ti_3_C_2_ and anti-EpCAM were included in the gelatin hydrogel. In its role as a cancer cell capture agent, anti-EpCAM improved the ensnaring of malignant cells. The cancer cells’ pseudopods were better able to grab the hydrophilic, rough surface of the modified hydrogel due to the hydrophilic feature of the Ti_3_C_2_ nanosheets. Gelatin hydrogel’s temperature-response capacity allows for weakening the adhesion between cancer cells and the hydrogel in response to thermal stimuli; these stimuli—either direct thermal stimuli or photothermal stimuli mediated by nanosheets under laser irradiation—could induce cell release for further analysis without damaging cancer cells (You et al., 2021).

Attributed to the nanosheet structure and rough surface, 2D materials are in favor of capturing cells. Though thermal stimuli are introduced into CTC release, the increase of PTT over direct thermal stimuli has not been identified. Regardless of generating heat during PTT, an excellent temporal and spatial resolution, which is unique to PTT mediated by 2D materials, has not been involved in CTC detection. In addition, the promotion of cell proliferation of PTT, that has been proven, is well worth considering in CTC detection. Given the existing problems in capturing CTCs, such as low concentration of CTCs in blood at an early stage of cancer and low viability of cells during detection, 2D-material-mediated PTT may be a significant complement to current strategies (Yoon et al., 2016). Different therapeutic strategies of PTT based on 2D materials are summarized in Table 1.

**TABLE 1:** Summary of different therapeutic strategies of PTT based on 2D materials.

Biomedical applications	Therapeutic modalities	2D substrates	Loadings	Functionalized materials	*In vivo* models	Ref
Cancer therapy	Single-mode PTT in NIR-I	BCN	—	HA@BCN	Melanoma-bearing BALB/c nude mice; C57 mice (back skin wound)	Zhao et al. (2022)
		BP (Xene)	—	BP–BG scaffold	Saos-2 tumor-bearing BALB/c nude mice; SD rats (cranial defect)	Yang B et al. (2018)
		Ti_3_C_2_ (MXene)	—	Ti_3_C_2_–BG scaffold	Saos-2 tumor-bearing BALB/c nude mice; SD rats (cranial defect)	Pan et al. (2020)
	Single-mode PTT in NIR-II	Nb_2_C (MXene)	—	Nb_2_C–PVP	4T1 tumor-bearing BALB/c nude mice	Lin et al. (2017)
		PtAg	—	SH-PEG-FA functionalized PtAg	4T1 tumor-bearing BALB/c nude mice	Zhang X et al. (2021)
		CaCuSi_4_O_10_ (X CuSi_4_O_10_)	—	CaPCu scaffold	143B tumor-bearing BALB/c nude mice; SD rats (calvaria defect)	He et al. (2021)
	PTT combined with drug therapy	Ti_2_N (MXene)	Doxorubicin/cisplatin	Ti_2_N@oSi	SMMC-7721 tumor-bearing BALB/c nude mice	Liu G et al. (2021)
		ZrC (MXene)	Prodrug SN38-Nif	ZrC@prodrug	MCF-7 tumor-bearing BALB/c nude mice	Liu Y et al. (2020)
	PTT combined with other therapeutic agent therapy	Ti_3_C_2_ (MXene)	Fe^2+^	Fe(II)-–Ti_3_C_2_	MKN45 tumor-bearing BALB/c nude mice	Wu et al. (2021a)
		Nb_2_C (MXene)	S-Nitrosothiol	MS/MXene-BG-SNO	Saos-2 tumor-bearing BALB/c nude mice; SD rats (cranial defect)	Yang Q et al. (2020)
	PTT combined with immunotherapy	BP (Xene)	R837	BP-PEG + R837	B16 tumor-bearing C57BL/6 mice	Wan et al. (2020)
		Pd (Xene)	Cytosine–phosphate–guanine	Pd(5)-CpG(PS)	B16F10 tumor-bearing C57BL/6 mice	Ming et al. (2020)
		FePSe_3_ (MPX_3_)	Anti-PD-1 peptide	FePSe_3_@APP@CCM	CT26 tumor-bearing C57BL/6 mice	Fang et al. (2021)
Bacterial infection therapy	Single-mode PTT	Ti_3_C_2_ (MXene)	—	Ti_3_C_2_ colloidal solution	KM mice (back *S. aureus* infection)	Wang et al. (2021a)
		Nb_2_C (MXene)	—	Nb_2_C@TP	BALB/c mice (upper dorsal *S. aureus* infection)	Yang et al. (2021)
	PTT combined with drug therapy	BP (Xene)	Kanamycin	Gal-BP@Kana	BALB/c mice (back PAO1 infected wound)	Guo et al. (2020)
		Ti_3_C_2_ (MXene)	Amoxicillin	MXene-AMX-PVA	BALB/c mice (back *S. aureus* infected wound)	Xu M et al. (2021)
	PTT combined with therapeutic agent therapy	BP (Xene)	ϵ-Poly-L-lysine	BP@ϵ-PL	ICR mice (dorsal *S. aureus* infection)	Fu et al. (2022)
		Polydopamine nanosheets	BNN6	GFPB hydrogel	BALB/c mice (upper back *S. aureus* infected wound)	Liu G et al. (2021)
Bone regeneration	PTT	BP (Xene)	—	BPs@PLGA	SD rats (tibial defect)	Tong et al. (2019)
		BP (Xene)	—	ZnL_2_–BPs@HAP	SD rats (tibial defect)	Wu et al. (2021b)
		MoS_2_ (TMDs)	—	PCL/MoS_2_	SD rats (tibial defect)	Ma T et al. (2021)
Neuronal modulation	PTT	Ti_3_C_2_ (MXene)	—	Ti_3_C_2_T_x_ films/Ti_3_C_2_ T_x_ flakes	—	Wang et al. (2021b)
Shape transformation	PTT	CaCuSi_4_O_10_ (XCuSi_4_O_10_)	—	CUP/PT	SD rats (IUA model)	Dong et al. (2022)
		BP (Xene)	—	PU/BP	BALB/c nude mice (implanted into the fallopian tube)	Xie et al. (2018)
Cellular uptake	PTT	BP (Xene)	Paclitaxel	BP-HAS-PTX	—	Wang et al. (2017)
		MoS_2_ (TMDs)	Cytosine–phosphate–guanine	MoS_2_–PEG–CpG	—	Han et al. (2017)
Cell detection	PTT	Ti_3_C_2_ (MXene)	—	AntiEpCAM-coated Ti_3_C_2_T_x_ @gelatin	—	You et al. (2021)

## 3 PDT

PDT is another form of non-invasive phototherapy that has minimal adverse effects and has recently gained more recognition (Sun L et al., 2021; Zhou et al., 2021). Photosensitizers (PSs) and light stimuli with a certain wavelength that coincides with the absorption spectra of PSs make up the core of the PDT mechanism. When PSs are exposed to suitable light irradiation, photooxidation is initiated through the Type I or Type II process, generating various ROS (^1^O_2_, ·OH, O, etc.) that have a strong oxidation capability and clearly damage target cells (Tsubone et al., 2019; Pang et al., 2020). PDT, which is attributed to the effects of ROS, has been used in treating cancer, disinfection, and skin diseases (Heinemann et al., 2017). However, organic compound PS flaws, such as lack of high water solubility and a mismatch between light excitation and deep tissue penetration, frequently limit the effectiveness of PDT (Abrahamse and Hamblin, 2017; Du and Tung, 2020). From this perspective, 2D materials with unique optical properties may be potential solutions for improving the therapeutic efficacy of PDT.

### 3.1 Single 2D material-mediated PDT

2D materials have been frequently used in PDT as PSs due to their potential bandgap and inherent structures. Due to their significant hydrophilicity, 2D materials are able to readily overcome the problems that arise with conventional PSs. PDT can be used with loading capacity and structural variation, significantly increasing the treatment efficacy (Wang X et al., 2020).

#### 3.1.1 PDT in cancer treatment

PDT has recently been used in clinical cancer therapy. Generated ROS can seriously harm biological components like lipids and nucleic acids, which have a clear cytotoxic effect on cancer cells (Bacellar et al., 2015). Additionally, ROS will damage the initial vasculature and impede the development of tumor arteries to obstruct blood supply to the tumor (Al-husein et al., 2004). Later, platelets are activated to block any residual blood arteries, which can prevent the tumor from absorbing nutrients (Nelson et al., 1987). Since BP has a high quantum yield within a wide wavelength absorption band and outstanding ROS production properties under laser irradiation, it has received the greatest attention from researchers studying photodynamics among innovative 2D materials used as PSs (Aksoy et al., 2020). It is noteworthy that BP contained DOX for synergistic cancer therapy. BP nanosheets demonstrated strong ROS production and excellent photostability following 606-nm laser irradiation at a low power density. In contrast, adding additional DOX had little impact on their photodynamic properties. The PDT–synergistic chemotherapy’s production of intracellular ROS and release of DOX demonstrated a positive anticancer effect (Chen Y et al., 2017).

**FIGURE 8:**
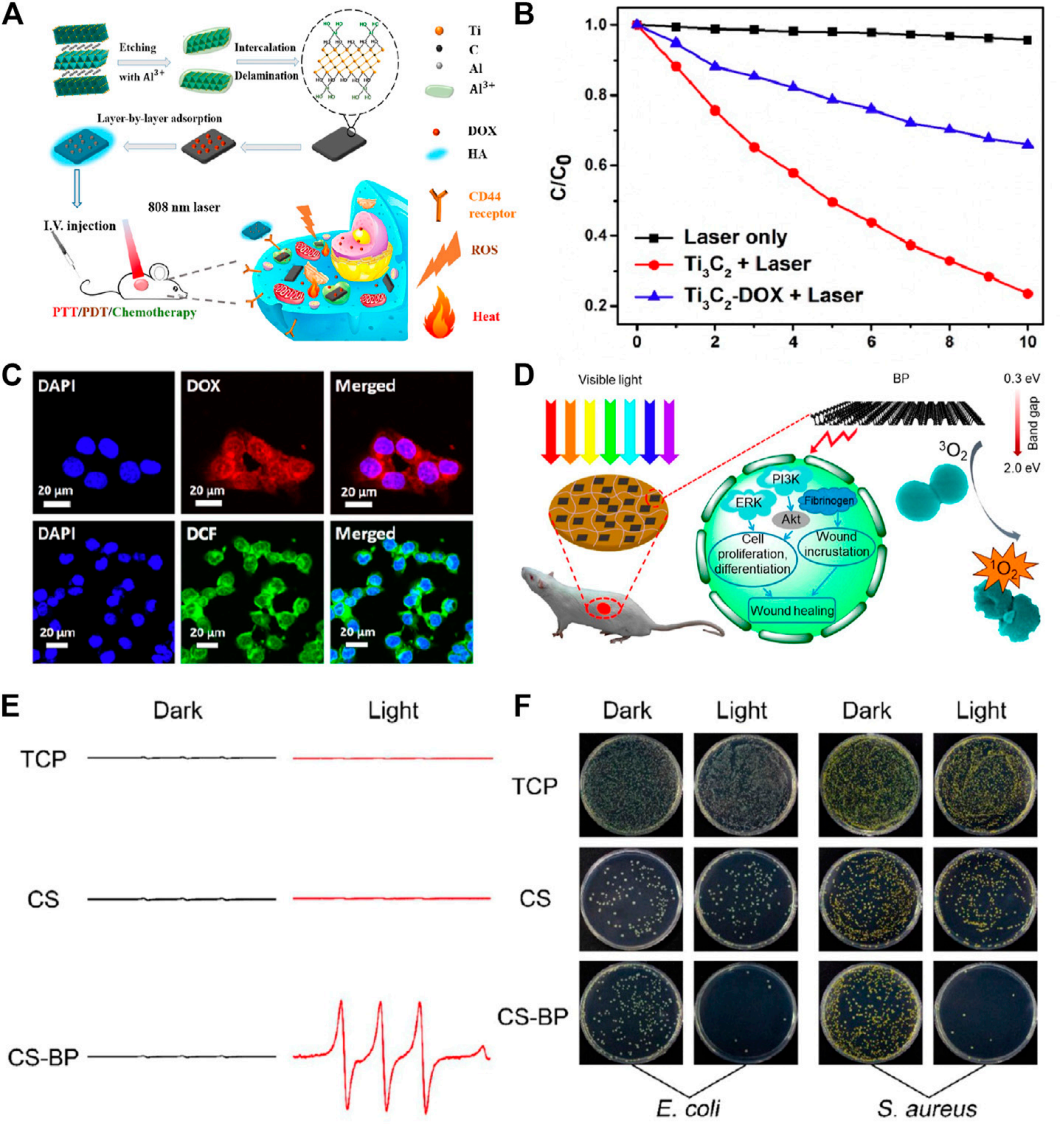
**(A)** Schematic illustration of fabrication of Ti3C2-DOX and therapeutic strategy of based on Ti3C2-DOX. **(B)** Absorbance of DCPF in different groups. **(C)** CLSM images of HCT-116c cells incubated with Ti3C2-DOX after irradiation. (Adapted with permission from Liu et al., 2017. Copyright 2017 American Chemical Society). **(D)** Schematic illustration of therapeutic strategy for bacterial infection and wound healing based on CS-BP. **(E)** ESP spectra in different groups. **(F)** Images of bacterial colony in different groups after treatments. (Adapted with permission from Mao et al., 2018. Copyright 2018 American Chemical Society).

Although UV and visible light are capable of producing ROS, its study in PDT is limited by the shallow tissue penetration depth. Accordingly, NIR photons with substantially higher penetration depth and very less biological tissue absorption may prevail in subsequent research (Yang et al., 2017; Wang X et al., 2020). Moreover, NIR lamps make it possible for synergistic phototherapy to be carried out under single-wavelength stimulation when PDT and PTT are combined (Jana et al., 2020). To achieve high stability and small flake size, which were essential to the permeability and retention effect of tumors, Ti_3_C_2_ MXene was modified by an additive Al^3+^ method (Figure 8A). Except for the photothermal behavior under 808-nm laser irradiation, ^1^O_2_ formation occurred at the same time in response to the same laser stimuli (Figure 8B). Numerous ROS were produced inside the cancer cells due to the cell absorption caused by nanosheets, which was a key factor in preventing tumor growth (Figure 8C) (Liu et al., 2017). Considering the similar structure to other nanosheets, the generation of ROS owed to the photoexcited electrons energy transferring from nanosheets to triplet oxygen (Wang et al., 2015). The LSPR effect, which has already been described in metal nanoparticles, may also contribute to the photodynamic capacity of MXene’s metal components (Jiang et al., 2013; Pasparakis, 2013). However, because of the lack of research on the photodynamic effect of MXene, the exact mechanism is still not clear, and the photodynamic strategy in tumor therapy and other biomedical applications remains to be studied.

In contrast, research on TMDs amply illustrated the photodynamic mechanism. TMDs showed exceptional photocatalytic properties among 2D materials due to a sufficient bandgap in a particular light region. Recent studies have found that TMDs in PDT have a promising photodynamic efficiency. Since heterostructures made from TMDs exhibit greater capacity in generating ROS, the potential of TMDs has not been fully realized. Using the cation-exchange approach to incorporate additional Bi atoms into freshly generated MoSe_2_ nanosheets, a specific heterostructure was created in MoSe_2_/Bi_2_Se_3_ nanosheets. Preservation of the electrons on Bi_2_Se_3_ and the photoinduced hole on MoSe_2_, which provided the nanosheets with an effective redox-active electron, would occur during laser irradiation. The photoinduced electron would shift from the conduction band of Bi_2_Se_3_ to the valence band of MoSe_2_. Additionally, concomitant PTT made it easier for charge transfer and heterostructure to coexist, which improved ROS formation and dramatically enhanced cancer cell apoptosis (Wang Y et al., 2019).

Similar to PTT, immunogenic cell death (ICD) generated by PDT also causes the release of DAMPs and TAAs, which facilitates the maturation of antigen-presenting cells and T-cell infiltration (Krysko et al., 2012; Li et al., 2018). Nanoplatforms, which carry immune adjuvants and immune checkpoint inhibitors to boost immune response, have recently been used in PDT-mediated immunotherapy. PDT immunotherapy for cancer treatment has produced excellent results with a number of nanoparticles (Wei et al., 2022). However, to the best of our knowledge, 2D materials have not yet been used in a synergistic method with tremendous potential for advancement in the treatment of cancer.

#### 3.1.2 PDT in bacterial infection treatment

Beyond cancer treatments, the photodynamic properties of 2D materials are also used in healthcare. Due to the fact that ROS has been shown to damage lipids and proteins in bacterial membranes, which leads to cell mortality, 2D materials have been used as an antibacterial treatment by utilizing their photodynamic properties (Xiang et al., 2019). On this subject, BP was studied in exploratory research (Figure 8D). After loading BP nanosheets onto chitosan hydrogel (CS), CS-BP revealed the generation of ^1^O_2_ after light illumination (Figure 8E). Attributed to the interaction between the negatively charged bacterial membrane and positively charged CS, CS partially destructed the bacterial membrane. Furthermore, with the additional photodynamic ability of BP, ROS production caused a destructive impact on bacteria after PDT, distinctly increasing the fatality of bacteria (Figure 8F) (Mao et al., 2018). The aforementioned combination demonstrated a strong curative effect for infectious injury with the help of PDT and the outstanding biocompatibility of hydrogel and nanosheets, while ROS did not show a noticeable slowing of wound healing.

As demonstrated in PTT, 2D materials with sharp edges caused physical damage to bacterial membranes. For weakening the side effects of ROS in PDT, such an antibacterial mechanism was fully utilized to support infection treatments, which could reduce the demand of ROS. An MXene-based antibacterial system was also reported. After a combination of modified MXene and Ag nanoparticles, the composite (M-HAS) at a low concentration exhibited satisfactory germicidal efficiency after 606-nm laser irradiation. The strong antibacterial effect benefitted from the generation of ROS and its sterilizing effect, which was the result of the destructive impact of Ag nanoparticles and the sharp edge of MXene flake on bacteria that formed the synergistic antibacterial therapy (Lv et al., 2022).

Another solution for the side effects of ROS is alleviating the negative impact of ROS on tissues. A metabolic intermediate 4OI was loaded onto BP nanosheets, which was further integrated with gelatin methacryloyl (GelMA). ROS generated by BP after laser irradiation distorted the bacterial structure, leading to exudation of the bacterial matrix. Later release of 4OI with prominent anti-inflammatory properties mitigated the damage of excessive ROS and facilitated the healing of injured tissue (Ding et al., 2022). Admittedly, 2D material-mediated PDT has exhibited high efficiency of ROS generation, and antibacterial effects already reached the requirements for most infections. As research in terms of enhancing bacteria killing is relatively mature, further investigation of PDT in bacterial infection tends to center on the healing process revolving around ROS scavenging.

### 3.2 2D materials combined with PSs in PDT

Another characteristic of 2D materials to be used in photodynamic treatment is being a nanocarrier loaded with organic PSs on account of their specific structure and optical property, which will increase the dispersibility and accumulation of PSs in target sites (Chen et al., 2020).

#### 3.2.1 2D materials combined with Ce6

As a commercial photosensitizer (PS), chlorin e6 (Ce6) has been applied not only in fluorescence imaging but also commonly used in photodynamic studies, parts of which include 2D materials (Sun J et al., 2021; Huang et al., 2022; Wang W et al., 2022). Polyethylene glycol (PEG)-modified BP nanosheet loaded with Ce6 was fabricated for tumor inhibition. With the help of the transfer function of the BP nanosheet, BP@PEG/Ce6 was absorbed into the cytoplasm and the intracellular level of Ce6 was promoted, which individual Ce6 could not reach. After laser irradiation, ROS would be generated inside the cancer cell, and the content was apparently increased with additional Ce6 loaded on BP. In addition, BP@PEG/Ce6 showed the slow release of Ce6 that was applicable for tumor hypoxic environments (Yang B et al., 2018). Such integration exploited the complementary effect of 2D materials and PSs that the cellular uptake of nanosheets and the strengthened photodynamic capacity of PSs multiplied the production of intracellular ROS. A similar delivery system also verified the prominent generation of intracellular ROS in the aforementioned integration as MoS_2_-PEG combined with Ce6 revealed the highest cancer cell killing efficiency after photodynamic treatment in contrast to single MoS_2_-PEG and single Ce6 (Liu et al., 2014).

Concerning antibacterial application, the research was focused on PDT with tissue selectivity. While ROS was generated in infectious tissues, PSs would also produce ROS in surrounding normal tissues, which is undesirable. In this regard, the selective release of PSs may be an option to improve matters. The MoS_2_ nanosheet carried with hyaluronic acid (HA)–Ce6 was investigated. With the presence of HA, Ce6 was kept nearby the MoS_2_ nanosheet in a normal physiological environment, which restricted the release of Ce6, making Ce6 ineffective under PDT. Upon reaching the infectious condition of MRSA filled with secretory hyaluronidase (HAase), HA would be degraded, resulting in a large release of Ce6, that further led to a higher generation of singlet oxygen under PDT. The HAase-responsive system tactfully avoids the harm caused by an ROS to normal cells, which may provide information for optimizing photodynamic therapy with accurate targets (Yuwen et al., 2021).

#### 3.2.2 2D materials combined with aggregation-induced emission PSs

Although Ce6 has been extensively applied in PDT with satisfactory results, a number of studies are still in progress aimed at finding alternatives for Ce6 and other organic PSs due to their common drawback, which is the aggregation-caused quenching effect that may hinder their biomedical application (Li L et al., 2021). Aggregation-induced emission (AIE) PSs are emerging to surmount the existing disadvantages of PSs over the past years. Contrary to the quenching effect in traditional PSs, AIE PSs reveal enhanced fluorescence under aggregate situations due to the limitation of intramolecular motion, which may expand the application of PSs (Chen K et al., 2021).

An AIE PS NH_2_-PEG-TTPy was used as a coating covering the BP nanosheet. Owing to the higher efficiency of intersystem crossing, BP@NH_2_-PEG-TTPy exhibited better yields of singlet oxygen than single Ce6. Through combination with PTT, ROS induced by white light stimuli and cellular uptake enhanced by NIR laser stimuli resulted in the prominent generation of intracellular ROS. In addition, on account of the EPR effect of BP@NH_2_-PEG-TTPy and the peculiarity of AIE that aggregates would induce intense emission, the obvious fluorescence signal of AIE was concentrated around the tumor, beneficial for fluorescence imaging (FLI) in cancer diagnosis (Huang H et al., 2020). Similarly, under laser irradiation, Ti_3_C_2_ loaded with AIE PS TBFT manifested significant ROS generation and strong tumor inhibition, synchronously revealing an overwhelming and sustainable fluorescence signal in the tumor site compared to the relatively weak and transient signal of conventional fluorochrome (Wang Y et al., 2022). In addition to combining the function of PSs for ROS production and fluorescent molecules for imaging, AIE PSs loaded on nanosheets also possess continuous fluorescence at target sites, which is crucial for long-term FLI navigation. It is undeniable that the combination of novel AIE PSs and 2D materials provided conducive insight into exploiting multimodal theranostic systems for cancer in which PDT is integrated with image guiding *via* novel PSs.

### 3.3 Improvements of PDT in the tumor microenvironment (TME)

Numerous studies have identified the significant prospects of PDT. However, several issues regarding the TME remain to be solved. Since the TME shows hypoxic conditions, the efficiency of PDT would be reduced as ROS generation relies on sufficient O_2_. In addition, overexpression of glutathione (GSH) in the TME serves as a scavenger of intracellular ROS and considerably restricts PDT in cancer treatments (Sun Y et al., 2021; Wang X et al., 2021).

#### 3.3.1 Manganese oxide

Noticeably, the manganese oxide (MnO_2_) nanosheet is currently under research to reverse hypoxia TME with excessive GSH. When reaching the acid environment at the tumor site, MnO_2_ would be reduced into Mn2+ and generate O_2_ through catalyzing a high content of H_2_O_2_, upgrading the oxygen level for PDT. In addition, GSH was consumed during the degradation of MnO_2_, which is in favor of sustaining ROS (Yang C et al., 2020; Xu Q et al., 2021).

MnO_2_ nanosheets were loaded with DNA-stabilized silver nanoclusters (AgNCs) and porphyrin (P) PS to construct the nanoplatforms (P-AgNCs-MnO_2_) with photodynamic activity. While under the TME, MnO_2_ would decompose due to the acid condition, promoting the release of P, which may ensure the effect of PS. Through degradation of MnO_2_, H_2_O_2_ would be converted into O_2,_ and the increased oxygen content could achieve a larger production of ^1^O_2_ under PDT, in which energy resonance transfer of remaining intact MnO_2_ also contributed. Meanwhile, the reaction between GSH and MnO_2_ lowered the GSH level, which protected ^1^O_2_ from being consumed by GSH, leading to a higher cancer cell fatality rate. In addition, Zn^2+^ released from AgNCs and degradation product Mn^2+^ could act as imaging agents for guiding therapy (Yao et al., 2019). The same result was demonstrated when the PS semiconducting polymer nanoparticle (SPN) was coated with MnO_2_. Under a hyperoxic environment induced by MnO_2_, the enhanced generation of ROS led to increased cancer cell necrosis, which further caused a significant suppressive effect on transplanted tumors (Zhu et al., 2018).

Showing degradation in a specific environment, MnO_2_ can serve as a switch in response to the TME, controlling the activation of PDT. Wrapped with MnO_2_, PS-modified gold nanoclusters (AuNCs@mSiO_2_) were concealed from light stimuli. In a normal physiological environment, the MnO_2_ shell blocked the reaction between internal PS and external stimuli, and PDT was turned off. While in the TME, MnO_2_ was degraded, turning on the switch of PDT and, in the meantime, producing enough O_2_ for ROS generation (Yin et al., 2021). The MnO_2_ nanosheet has been favored for its TEM-responsive capacity, which mediates O_2_ generation and GSH downregulation, easily reversing hypoxia in PDT for cancer. MnO_2_ may thus be a promising alternative nanocarrier to current PS-loaded 2D materials limited in tumor therapies. The toxicity and biocompatibility of MnO_2_ need to be further studied as the liberation of metal ions (Mn^2+^) remains a substantial concern (Huo et al., 2020).

#### 3.3.2 Photosynthesis

Photosynthesis is another solution for the TME by upregulating oxygen levels. Since there is abundant water in the human tissue, water is becoming the potential raw material for generating oxygen in a hypoxic environment. Compared to previous strategies for yielding oxygen, water emerges with nearly unlimited production of O_2_ and higher biosafety. Based on the splitting of water, several plants, bacteria, and nanomaterials have been exploited as photosynthesis agents in PDT to produce oxygen. Nevertheless, more research has yet to be carried out in combining photosynthesis with 2D materials in PDT (Huo et al., 2020; Li W et al., 2020; Cheng et al., 2021; Liu G et al., 2021).

Cyanobacterium *S. elongatus* was combined with BP nanosheets to reverse the hypoxia of the TME innovatively. Under 606-nm laser irradiation, water was converted into oxygen through photosynthesis induced by bacterial cells. Meanwhile, the transportation of oxygen outside the bacterial cells took place. Increased oxygen content reversed the hypoxia condition, distinctly enhancing the generation of ^1^O_2_ in cancer cells after irradiation. In the presence of cyanobacteria, BP-mediated PDT revealed a strengthened ability of tumor inhibition. Furthermore, bacteria showed no adverse effect on the organisms, illustrating the significant validity and biosecurity of bacteria-mediated photosynthesis in PDT for tumor eradication (Qi et al., 2021). Except for the listed method for confronting the TME, certain catalase-like agents have also been applied combined with nanocarriers to overcome hypoxia conditions (Jannesari et al., 2020; Ran et al., 2021). However, no standard strategy for the TME has been achieved at present, still having a long way to go before optimizing PDT in the TME.

### 3.4 2D material-based PDT-chemodynamic therapy

Despite the gratifying curative effect and negligible side effects of PDT, the inability of entire tumor eradication through a single PDT provides the possibility of further research in multimodal therapy based on ROS. Chemodynamic therapy (CDT) in which endogenous H_2_O_2_ could be converted into ·OH through Fenton or Fenton-like reactions induced by metal-based agents may be a potential measurement to enhance ROS production combined with PDT (Li L et al., 2020; Zhu et al., 2022). As mentioned in PTT, chemodynamic agents have been applied loaded on nanosheets. Early research demonstrated the enhanced ROS generation of PDT–CDT synergistic therapy that chemodynamic agent FePt nanoparticles loaded on BP could spontaneously produce additional ROS during the treatment (Yao et al., 2020). Different from carrying chemodynamic agents in the previous studies, 2D materials being chemodynamic agents themselves are recently gaining attention.

In this context, FeWO_X_–PEG was fabricated for tumor inhibition after the modification of amphiphilic polymer on iron tungsten oxide nanosheets through organic-phase synthesis. Under the acid situation of the TME, Fe^2+^ and Fe^3+^ are easily released owing to the distinct structure of the **n**anosheet, which makes iron atoms disperse throughout the surface. Extra Fe^2+^ was formed within the reaction between Fe^3+^ and GSH after cell uptake into the lysosome. Later, H_2_O_2_ was reduced to ·OH consuming Fe^2+^ through the Fenton reaction in which ·OH would further destroy the lysosome, leading to the escape of FeWO_X_–PEG to the cytoplasm. PDT was then performed under 1060-nm laser irradiation, causing the generation of intracellular ^1^O_2_. Moreover, while combining PDT with CDT, marked improvement in ROS production ultimately enhanced tumor inhibition (Xiang et al., 2022). LDHs nanosheets in another study exhibited a similar role as FeMn–LDH could function as a chemodynamic agent through releasing Fe^3+^ and Mn^2+^. With the assistance of loading Ce6 and up-conversion of nanoparticles, the PDT effect of FeMn–LDH was enhanced, resulting in a nearly complete eradication of the tumor after PDT/CDT synergistic treatment (Jia et al., 2020).

Although PDT/CDT synergistic therapy is common in various studies, mediating PDT and CDT through a single agent is relatively new in tumor therapy. The investigations significantly expand the application range of 2D materials, which may pave the path for study toward a fresh strategy of multimodal tumor therapy. Nevertheless, the biocompatibility of released metal ions needs to be tested with additional research. In addition, combining photothermal and photodynamic capacity to form synergistic phototherapy is also common in various research studies on anticancer and antibacterial therapy that 2D materials are regarded as desired agents for phototherapy. Different therapeutic strategies of PDT based on 2D materials are summarized in Table 2.

**TABLE 2:** Summary of different therapeutic strategies of PDT based on 2D materials.

Biomedical applications	Therapeutic agents	2D substrates	Functionalized materials	Therapeutic benefits	*In vivo* models	Ref
Cancer therapy	Single 2D materials	BP (Xene)	BP-DOX	Synergistic PDT/PTT/chemotherapy enhancing treatment efficiency	4T1 tumor-bearing BALB/c mice	Chen Y et al. (2017)
		Ti_3_C_2_ (MXene)	Ti_3_C_2_-DOX	Synergistic PDT/PTT/chemotherapy (single-wavelength laser activation) enhancing treatment efficiency	HCT-116 tumor-bearing athymic nude mice	Liu et al. (2017)
		MoSe_2_ (TMDs)	MoSe_2_/Bi_2_Se_3_	Heterostructure enhancing PDT	U14 tumor-bearing KM mice	Wang Y et al. (2019)
		FeWOx (TMOs)	FeWOx-PEG	Synergistic PDT/PTT/CDT/immunotherapy enhancing treatment efficiency	4T1 tumor-bearing BALB/c nude mice	Xiang et al. (2022)
	2D materials combined with Ce6	BP (Xene)	BP@PEG/Ce6	Synergetic action of PSs and nanosheets enhancing PDT	HeLa tumor-bearing nude mice	Yang B et al. (2018)
		MoS_2_ (TMDs)	MoS_2_-PEG/Ce6	Synergetic action of PSs and nanosheets enhancing PDT	4T1 tumor-bearing BALB/c mice	Liu et al. (2014)
		FeMn-LDH (LDHs)	UCSP-LDH	Synergistic PDT/PTT/CDT enhancing treatment efficiency	U14 tumor-bearing KM mice	Jia et al. (2020)
	2D materials combined with AIE PSs	BP (Xene)	BP@NH_2_-PEG-TTPy	Fluorescence imaging for guiding treatment	4T1 tumor-bearing BALB/c nude mice	Huang H et al. (2020)
		Ti_3_C_2_ (MXene)	TBFT, UCNP@DSPE-PEG@ Ti_3_C_2_	Continuous and strong fluorescence signal for guiding treatment	4T1 tumor-bearing BALB/c mice	Wang L et al. (2020)
	2D materials combined with porphyrin	MnO_2_ (TMOs)	P-AgNCs-MnO_2_	Reversing the TME enhancing PDT	MCF-7 tumor-bearing nude mice	Yao et al. (2019)
	2D materials combined with SPN	MnO_2_ (TMOs)	SPN-Ms	Reversing the TME enhancing PDT	4T1 tumor-bearing BALB/c mice	Zhu et al. (2018)
	2D materials combined with AuNCs	MnO_2_ (TMOs)	AuNCs@mSiO_2_MnO_2_@	Intelligent PDT/reversing the TME enhancing PDT	MDA-MB-435 tumor-bearing Nu/nu mice	Yin et al. (2021)
	2D materials combined with cyanobacterium	BP (Xene)	Cyan@BPNSs	Reversing the TME enhancing PDT	4T1 tumor-bearing BALB/c mice	Qi et al. (2021)
Bacterial infection therapy	Single 2D materials	BP (Xene)	CS-BP	Prominent biocompatibility promoting wound healing	Wistar rats (back *S. aureus* infected wound)	Mao et al. (2018)
		Ti_3_C_2_ (MXene)	M-HAS	Dual antibacterial mode enhancing treatment efficiency	-	Lv et al. (2022)
		BP (Xene)	4OI-BP@Gel	Anti-inflammatory action promoting wound healing	SD rats (back infected wound)	Ding et al. (2022)
	2D materials combined with Ce6	MoS_2_ (TMDs)	MoS_2_@HA-Ce6	Intelligent PDT/PTT enhancing treatment efficiency	BALB/c mice (thigh *MRSA* infected wound)	Yuwen et al. (2021)

## 4 Summary and outlook

This review offered a comprehensive analysis of many unique 2D materials used in standard phototherapy. The use of 2D materials in anticancer and antibacterial therapies under both NIR-I and NIR-II stimuli is of tremendous academic interest due to their excellent light absorption in broad bio-windows and high photothermal conversion efficiency. Additionally, a particular composition and nanosheet structure are being investigated in combination with PTT to create a nanoplatform that can be used for loading drugs and therapeutic agents, immunotherapy, bone regeneration, neuronal modulation, shape transformation, cellular uptake, and cell detection. The dual characteristics of 2D materials used in PDT are illustrated, along with strategies for boosting PDT effectiveness under the TME. Due to their 2D structure and superior optical properties, 2D materials are specifically used as PS carriers and prospective organic PS substitutes. They are also proven to increase ROS formation during CDT and reverse the TME during PDT by utilizing certain chemical components.

It is noteworthy that compared to other nanomaterials, 2D materials possess specific advantages conducive to various biomedical applications. 1) 2D structure provides nanosheets with a large surface-to-volume ratio, which endows them with an excellent functionalization ability for enhancing dispersity and stability in a physiological environment. Meanwhile, abundant active sites on the large surface enable the easy anchoring of versatile therapeutic agents on nanosheets. 2) The optical property of nanosheets is highly controllable *via* the adjustment of the thickness of nanosheets, which is beneficial to enhancing photothermal and photodynamic abilities. The unique properties contribute to the broad application of 2D materials. In addition to the mentioned therapeutic strategies, image guiding for phototherapy also shows fascinating prospects in applying 2D materials. The development of 2D materials as contract agents to improve bioimaging performance, namely, for computed tomography (CT), fluorescence imaging, photoacoustic imaging, and magnetic resonance imaging, is due to their better photoelectric properties (Soleymaniha et al., 2019). In addition, ascribed to the large-area immobilization of sensing targets and the fast electron transfer fluorescence-quenching effect, 2D materials have been employed as prominent alternatives to traditional nano-biosensors for detecting biomacromolecules and biological processes (Lin et al., 2018; Lam et al., 2021). The novel biosensing system may provide the platform for tracing therapeutic effects in clinics with high selectivity and efficiency. In this respect, the contrast-enhanced image and biosensors based on 2D materials can further facilitate the diagnostic image guidance and monitor phototherapy and corresponding body response, forming a multifunctional theragnostic platform for various diseases.

Despite the significant advancements in 2D materials that have been made, several problems and difficulties that impede clinical translation still need to be resolved. One of them is that top–down approaches and bottom–up methods both have their flaws, making it difficult to strike a balance between yield, size, shape, and purity, which determine the property of 2D materials. Another significant limitation is long-term chronic biosafety, which makes it difficult to assess a material’s biocompatibility due to the variability in synthetic environments, modifications, and experimental conditions. Furthermore, even under mild hyperthermia, side effects caused by generated heat and ROS are unavoidable, which may delay the treatment. Though 2D materials have progressed in precise treatment, subcellular localization still needs to be optimized. Additionally, penetration depth also restricts their development. Currently, NIR-I still dominates in the phototherapy of 2D materials, lacking deep tissue penetration capacity. In addition, a study in NIR-II is limited to 1064-nm laser irradiation, while light with other wavelengths is not widely exploited yet. To compare various kinds of 2D materials, the existing study is limited to the effect of constituent elements in tissue engineering. The elemental influence on other properties of 2D materials related to phototherapy for cancer, bacterial infection, and other diseases has not been investigated, and further study is needed for detecting therapeutic actions of distinct nanosheets in different diseases.

In terms of the phototherapy mediated by nanoagents, 2D materials have shown distinct advantages as large surface areas form the drug delivery system, sharp edges enhance antibacterial function, unique components and mechanical flexibility promote tissue regeneration, and more, which strengthen the effect of phototherapy for various diseases. However, the investigation of phototherapy is not confined to 2D materials. Recently, plasmonic nanoparticles have demonstrated extraordinary performance in phototherapy. Except for the excellent photoelectric property comparable to that of 2D materials, nanoparticles with tunable size and shape and abundant surface-active sites can provide approaches to more modifications that are highly regioselective and stereoselective, beneficial to the adjustment and improvement of photoelectric properties. In addition, the tiny size in three dimensions and outstanding optical properties can further enhance the spatiotemporal control of phototherapy and the accuracy of image guidance (Tyagi et al., 2020; Chang et al., 2021; Yin et al., 2023). Hence, further study is required to combine 2D materials with other nanomaterials to fully utilize the advantages and compensate for the shortages of each nanomaterial.

In conclusion, 2D materials have generated much attention in the biomedical sector recently, with phototherapy showing the most promise for future research. Although it is challenging to create a single 2D material appropriate for all forms of phototherapy, it is anticipated that several 2D materials will be chosen, and their features will be tuned to meet certain clinical needs in later research. However, we are optimistic that the evaluation will be very helpful and improve future research on 2D materials in phototherapy.
